# Controllable synthesis of ZnO nanostructures on the Si substrate by a hydrothermal route

**DOI:** 10.1186/1556-276X-8-378

**Published:** 2013-09-05

**Authors:** Jing-Jing Dong, Chun-Yang Zhen, Hui-Ying Hao, Jie Xing, Zi-Li Zhang, Zhi-Yuan Zheng, Xing-Wang Zhang

**Affiliations:** 1 School of Science, China University of Geosciences, Beijing 100083, China; 2 Key Lab of Semiconductor Materials Science, Institute of Semiconductors, Chinese Academy of Science, Beijing 100083, China

**Keywords:** Zinc oxide, Nanostructure, Hydrothermal

## Abstract

In this paper, controllable synthesis of various ZnO nanostructures was achieved via a simple and cost-effective hydrothermal process on the Si substrate. The morphology evolution of the ZnO nanostructures was well monitored by tuning hydrothermal growth parameters, such as the seed layer, solution concentration, reaction temperature, and surfactant. X-ray diffraction and photoluminescence measurements reveal that crystal quality and optical properties crucially depend on the morphology of the ZnO nanostructures. The ease of synthesis and convenience to tune morphology and optical properties bring this approach great potential for nanoscale applications.

## Background

ZnO nanostructures have attracted extensive attention over the past few years because of their unique properties for applications in electronic and optoelectronic devices [1-5]. For example, by virtue of the nanosized junction and excellent waveguiding property of nanorods, the ZnO nanorod-based heterojunction light-emitting diodes (LEDs) exhibit significantly improved electroluminescence performance [6-8]. It is well known that the properties and applications of ZnO are crucially dependent on the microstructures of the materials, such as morphology, size, and orientation. Hence, controllable synthesis of ZnO nanostructures is of great importance to tailor their physical properties and improve device performance [9-11]. So far, ZnO nanostructures have been synthesized by various physical and chemical methods, such as vapor–liquid-solid, molecular beam epitaxy, and solution processes. Among them, room temperature solution route (hydrothermal method, for example) is particularly attractive because it is a simple, low-temperature, and catalyst-free process with no limitation of substrates [1,12-15]. In addition, by varying the reaction parameters during hydrothermal process, morphology of ZnO nanostructures can be tuned effectively [16]. In this paper, controllable synthesis of various ZnO nanostructures on the Si substrate was achieved by tuning hydrothermal growth parameters, such as the seed layer, solution concentration, reaction temperature, and surfactant. X-ray diffraction (XRD) and photoluminescence (PL) measurements reveal that crystal quality and optical properties crucially depend on the morphology of the ZnO nanostructures.

## Methods

### Deposition of ZnO seed layers on the Si substrates

Here, ZnO seed layers were prepared by two methods: radio-frequency (RF) magnetron sputtering and dip coating, as described in the following.

#### RF magnetron sputtering

The ZnO seed layer was deposited on Si substrates by a conventional RF magnetron sputtering system equipped with a ZnO (99.99%) ceramic target. The sputtering chamber was evacuated to a base pressure of 1.0 × 10^−5^ Pa and then filled with working gas (pure Ar) to a pressure of 1.0 Pa. After depositing at 600°C with a constant RF power of 80 W for certain time intervals, a layer of ZnO nanoparticles was obtained.

#### Dip coating

Zinc acetate dihydrate (Zn(CH_3_COO)_2_ · 2H_2_O) and monoethanolamine (C_2_H_7_NO) were firstly dissolved in ethanol with an identical concentration of 0.05 M. Then, the solution was stirred at 60°C for 5 min to yield a clear and homogeneous solution. Next, a clean Si substrate was dipped into the solution, lifted at 1 mm/s, and dried in the air. Finally, the as-coated substrate was sintered at 250°C for 10 min to achieve ZnO seed layers [1,17].

### Hydrothermal growth of ZnO nanorods

To grow ZnO nanostructures, the Si substrates coated with the ZnO seed layers were fixed upside down in the reaction vessel containing 40 ml of aqueous solution of Zn(NO_3_)_2_ ⋅ 6H_2_O (99.5% purity, Sigma-Aldrich Corporation, St. Louis, MO, USA) and hexamethylenetetramine (99.5% purity, Sigma-Aldrich) with the identical concentration. Then, the reaction vessel was sealed and kept at a constant temperature for a certain time. Finally, the sample was taken out, rinsed in deionized water, and dried in air for characterization [18].

### Characterization

Surface morphologies of the seed layers and ZnO nanostructures were characterized by atomic force microscopy (AFM; Solver P47, NT-MDT, Moscow, Russia) and field-emission scanning electron microscopy (SEM; FE-S4800, Hitachi, Tokyo, Japan), respectively. The crystal structure identification of the ZnO nanostructures was performed by XRD in a normal *θ*-2*θ* configuration using a Rigaku (Tokyo, Japan) Dmax 2500 diffractometer with a Cu Kα X-ray source. The PL spectra were acquired by excitation with a 325-nm He-Cd laser with a power of 30 mW at room temperature.

## Results and discussion

For hydrothermal growth of ZnO nanostructures on lattice-mismatched substrates, such as the Si substrate, the ZnO seed layer is essential [19,20], which will influence the morphology and orientation of resulting ZnO nanostructures. Thus, we investigate the effect of deposition method and thickness of the seed layer on the ZnO nanostructures in the following.

**Figure 1:**
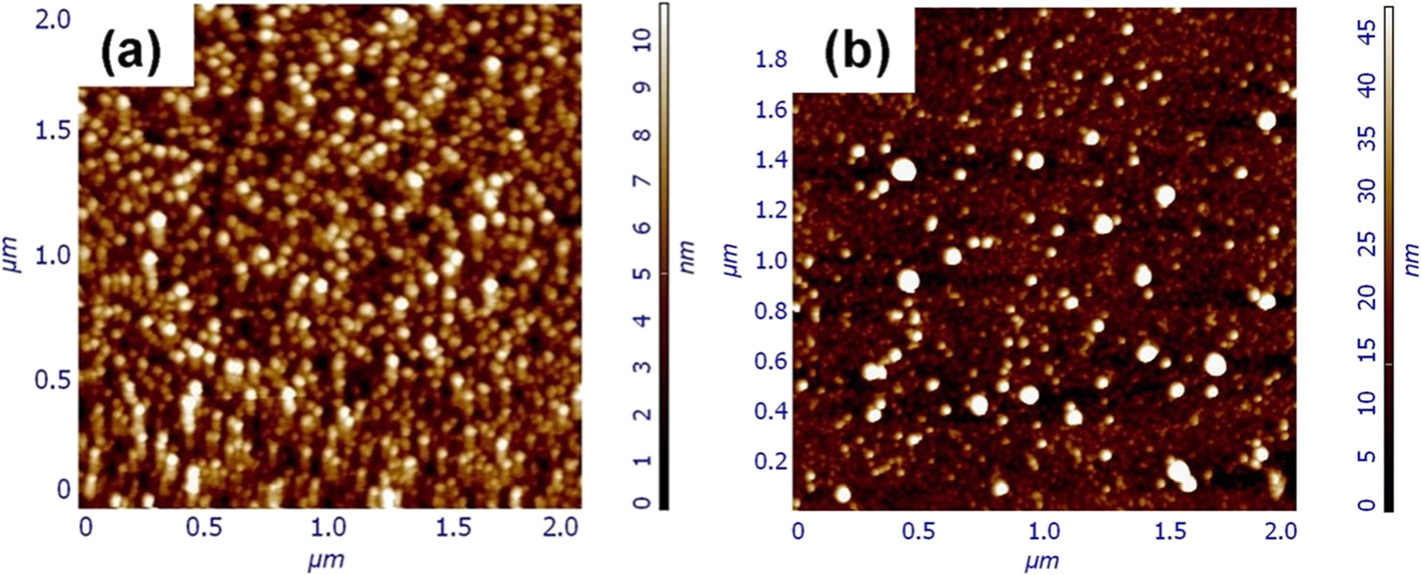
**AFM images of ZnO seed layers.** They are prepared by **(a)** RF magnetron sputtering (40 nm in thickness) and **(b)** dip coating.

The typical AFM images of the ZnO seed layers prepared by RF magnetron sputtering and dip coating are shown in Figure 1a,b, respectively, to distinguish typical surface features previous to the hydrothermal process. It is obvious that the size and roughness of the seed layers by different methods vary widely. Both ZnO seed layers present a high density of ZnO seeds, which act as nucleation sites during the growth step, and will decide the density of resulting ZnO nanostructures [21]. In addition, the size and roughness of the seed layer also have a significant effect on the growth mode and morphology of the ZnO nanostructures [22]. The diameter and root-mean-square (rms) roughness of seed layers can be derived from the AFM data corresponding to the AFM images shown in Figure 1a,b. For seed layers deposited by RF magnetron sputtering and dip coating methods, the corresponding diameter of seeds is 25 to 35 nm and 40 to 90 nm, and the rms roughness is 1.17 and 4.28 nm, respectively. From these results, it is found that the size distribution and surface roughness of the seed layer prepared by dip coating are much larger than that prepared by RF magnetron sputtering method, which is common for seed layers deposited by a solution process and would have a bad effect on the resulting ZnO nanostructures (see below) [23].

**Figure 2:**
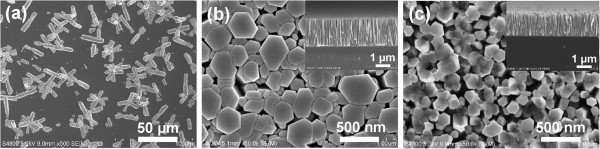
**SEM images of ZnO nanostructures.** They are grown on **(a)** bare Si substrate, the Si substrate coated with the seed layer deposited by **(b)** RF magnetron sputtering (40 nm in thickness) and **(c)** dip coating, at 0.05 M, at 95°C for 5 h (insets are corresponding cross-sectional images).

Figure 2a,b,c shows the SEM images of ZnO nanostructures grown on bare Si substrate, on the Si substrate coated with seed layer deposited by RF magnetron sputtering (40 nm in thickness), and on the Si substrate coated with seed layer deposited by dip coating method, respectively, at 0.05 M, at 95°C for 5 h. As can be seen, there are ZnO nanostructures grown on all of the three substrates. Among them, there are randomly oriented ZnO nanoflowers at low density on the bare Si substrate, as shown in Figure 2a. Without the seed layer, the nucleation density is remarkably lower than that grown with seeds because nucleation of ZnO nanostructures on seeds has a lower free energy barrier of activation than on the bare Si substrate [9]. In contrast, Figure 2b,c presents that ZnO nanorods grown on the Si substrate coated with the seed layer deposited by RF magnetron sputtering and dip coating are *c*-axis-oriented at high density, indicating that the seed layer plays an essential role in promoting nucleation and guiding oriented growth. Especially, the nanorods grown on the RF-sputtered seed layer is perfectly aligned normal to the substrate with uniform height, which is due to the low roughness and even distribution of the RF-sputtered seed layer, while the broad size distribution and large surface roughness of the dip-coated seed layer lead to poor orientation and surface roughness of the ZnO nanorods as shown in Figure 2c, which will be further confirmed by the following XRD measurement.

**Figure 3:**
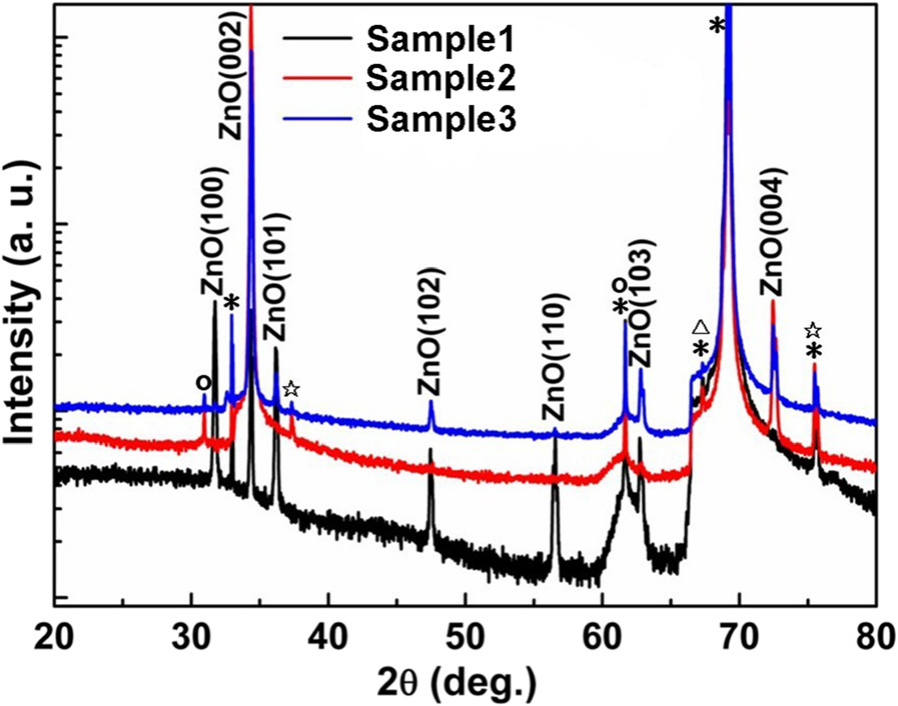
**XRD patterns of the ZnO nanostructures.** They are grown on the bare Si substrate (sample 1), RF-sputtered (sample 2), and dip-coated (sample 3) seed layers in a *θ*-2*θ* configuration (* peaks from the Si substrate; o, ☆, and △ are non-monochromaticity of the X-ray source induced by K_β_, Ni, and W, respectively).

The crystal structure on the ZnO nanostructures grown on bare Si substrate (sample 1), RF-sputtered seed layer (sample 2), and dip-coated seed layer (sample 3) was studied using XRD measurements in a *θ*-2*θ* configuration, as shown in Figure 3. Except for the peaks caused by the Si substrate and the non-monochromaticity of the X-ray source, the XRD patterns of the three samples share two peaks at 34.44° and 72.56°, corresponding to ZnO (002) and (004), respectively. The absence of any other peaks from the XRD pattern of sample 2 within the experimental resolution indicates the high *c*-axis orientation of ZnO nanostructures grown on RF-sputtered seed layer. Although the ZnO (002) peak dominated, weak ZnO (101), (102), and (103) peaks were observed clearly in the XRD pattern of sample 3, implying the poor *c*-axis orientation of ZnO nanostructures grown on dip-coated seed layer. From the XRD pattern of sample 1, we can see that ZnO (100), (002), (102), (110), and (103) peaks appear at about the same intensity, demonstrating the random orientation of ZnO nanostructures grown on the bare Si substrate [14,21]. Conclusions drawn from the XRD patterns are in high accordance with those drawn from earlier SEM results.

**Figure 4:**
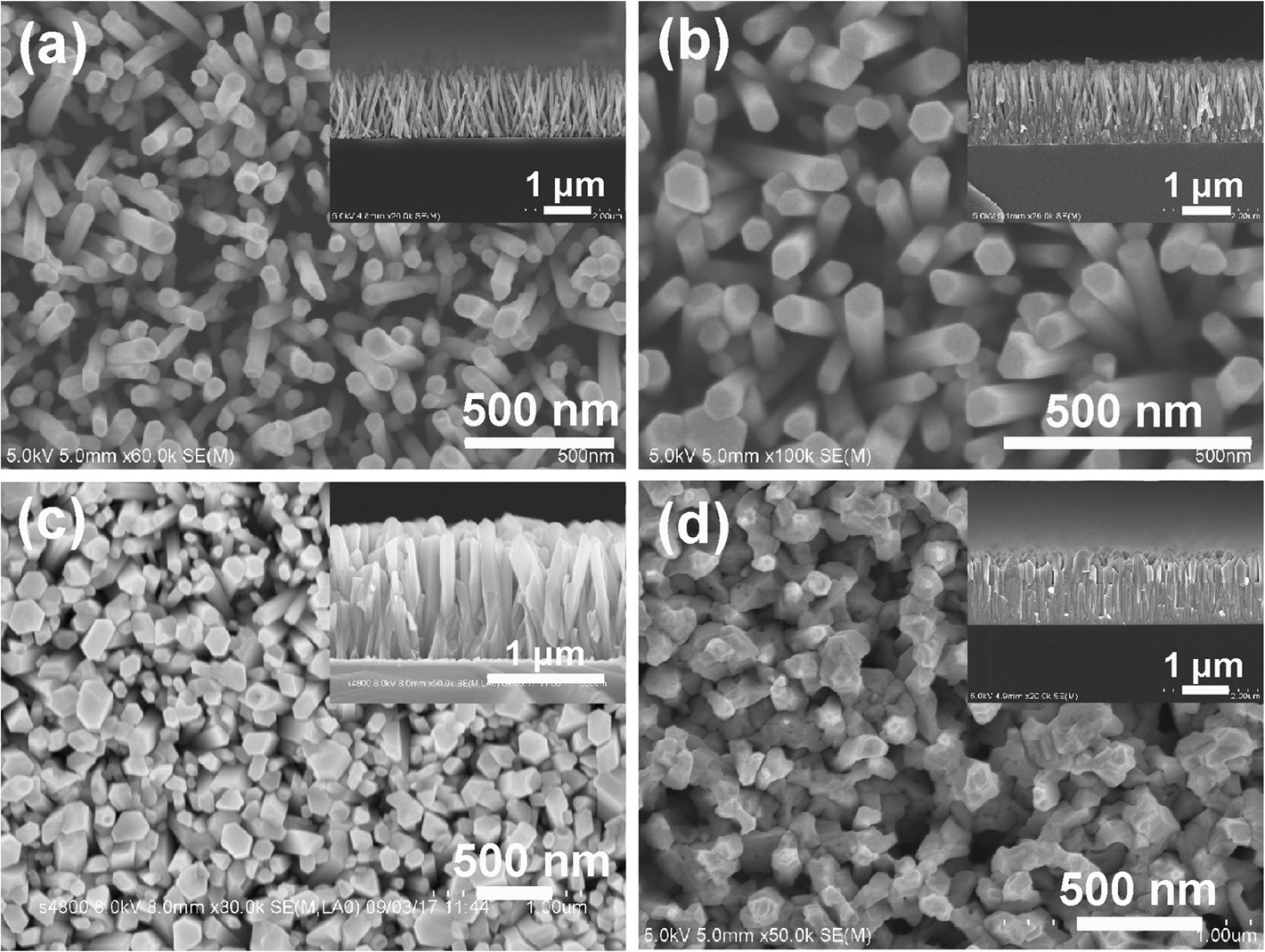
**Plan view and cross sections (insets) SEM images of the ZnO nanorods.** They are grown at 0.025 M, at 85°C for 5 h on the RF-sputtered seed layer with a thickness of **(a)** 40 nm, **(b)** 80 nm, **(c)** 300 nm, and **(d)** 1 μm, respectively.

As mentioned above, ZnO nanorods grown on RF-sputtered seed layer have high *c*-axis orientation and uniform height, which are attributed to the low roughness and even size distribution of the seed layer. However, it is reported that the roughness and size distribution vary with the thickness of the seed layer [23], so hydrothermal growths of ZnO nanorods on RF-sputtered seed layers with different thicknesses are performed. Figure 4a, b, c, d shows the plan view and cross section (insets) of the ZnO nanorods grown at 0.025 M, at 85°C for 5 h, on the RF-sputtered seed layer with thickness of 40, 80, 300 nm, and 1 μm, respectively. It is known that the size of ZnO seeds increases with the sputtering time, so the larger in thickness, the larger is the size of seeds. Actually, when the thickness increases to a certain value, the seeds will connect with each other and become a film. Besides, the seeds play an important role in inhibiting the ZnO nanorods from lateral growth, and smaller seeds yield thinner nanorods [23,24]. As a result, the diameter of ZnO nanorods increases with the thickness of the seed layer, as shown in Figure 4. In addition, it is obvious that the ZnO nanorods grown on 40- and 80-nm seed layers are inclined but become perfectly aligned normal to the substrate when the thickness increases to 300 nm, which is due to the improved crystal quality of the seed layers as the sputtering time increases.

**Figure 5:**
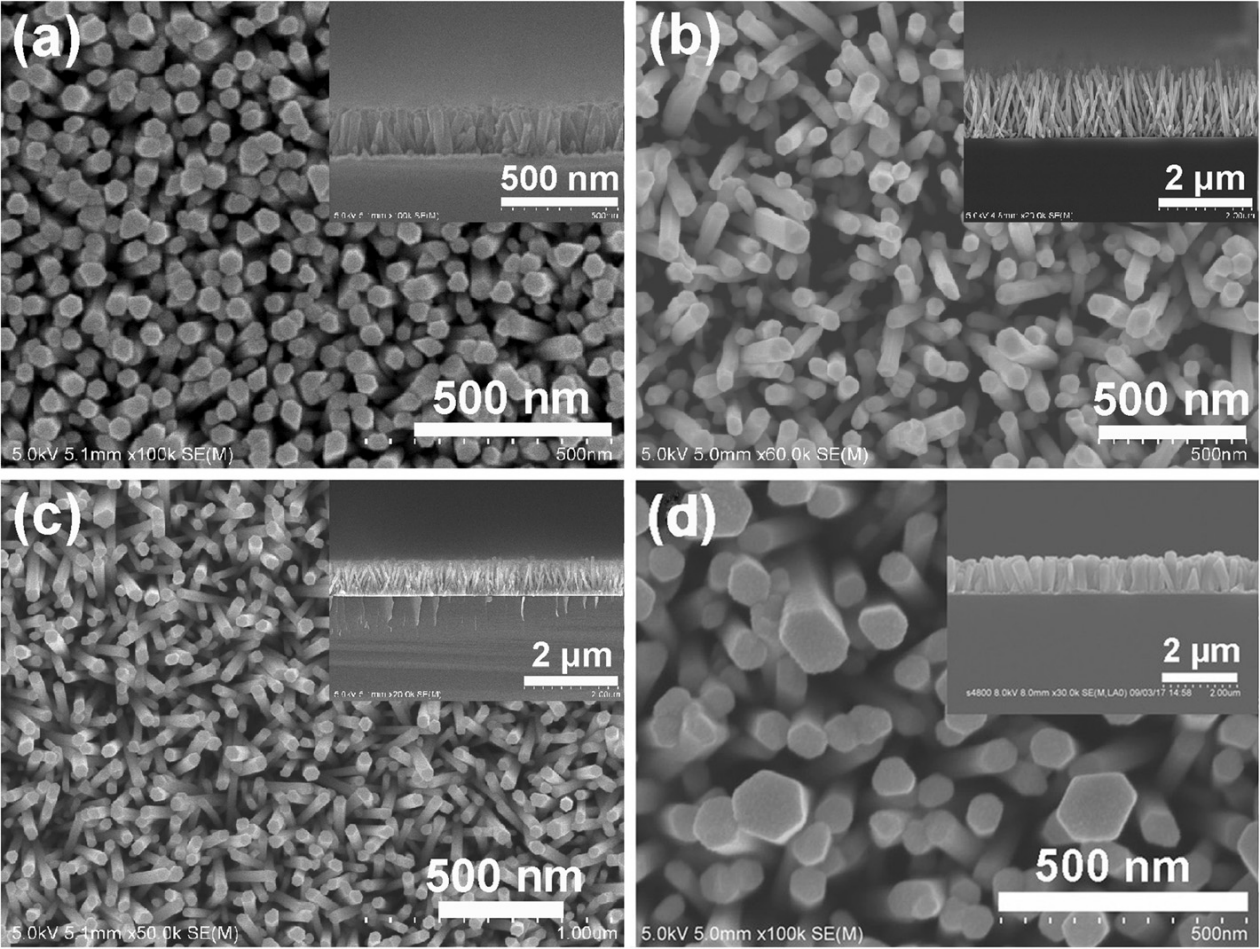
**Plan-view and cross-sectional (insets) SEM images of ZnO nanorods obtained at different temperatures and concentrations.** Temperatures **(a)** 60°C and **(b)** 85°C at a concentration of 0.025 M for 5 h; concentrations of **(c)** 0.01 M and **(d)** 0.03 M at 80°C for 5 h.

Hydrothermal growth of ZnO nanostructures is a chemical process, so the reaction temperature and solution concentration are two critical parameters, which will affect the reaction rate and then the morphology of ZnO nanostructures. Thus, we studied the influence of the reaction temperature and solution concentration on the ZnO nanorods in the following. Figure 5a,b shows the plan-view and cross-sectional SEM images of ZnO nanorods prepared at temperatures of 60°C and 85°C, respectively while keeping the solution concentration (0.025 M) and reaction time (5 h) constant. It can be seen that the diameters of ZnO nanorods grown at different temperatures are almost identical (about 50 nm), but their lengths vary greatly: the length of the nanorods increases rapidly from 300 nm to 1.5 μm, with the increasing reaction temperature from 60°C to 85°C, as shown in the insets of Figure 5a, b. Hence, the growth rate along the *c*-axis will be much faster than the radial direction, as the reaction temperature increases. Figure 5c, d shows the plan-view and cross-sectional SEM images of ZnO nanorods synthesized at different concentrations (0.01 and 0.03 M) while keeping the temperature (80°C) and deposition time (5 h) constant. In contrast with the results with different temperatures, the diameter of ZnO nanorods grown at different concentrations varies greatly from about 35 to 70 nm as the solution concentration increases from 0.01 to 0.03 M. Compared with the diameter, the difference in length is much smaller, and the lengths of the nanorods synthesized at 0.01 and 0.03 M are 0.9 and 1.0 μm respectively, as shown in the insets of Figure 5c, d. Hence, the growth rate along the radial direction will be much faster than that in the *c*-axis as the solution concentration increases, as reported in previous reports [25,26]. Above all, the length of ZnO nanorods depends mainly on the reaction temperature, while the diameter is closely related to the solution concentration.

**Figure 6:**
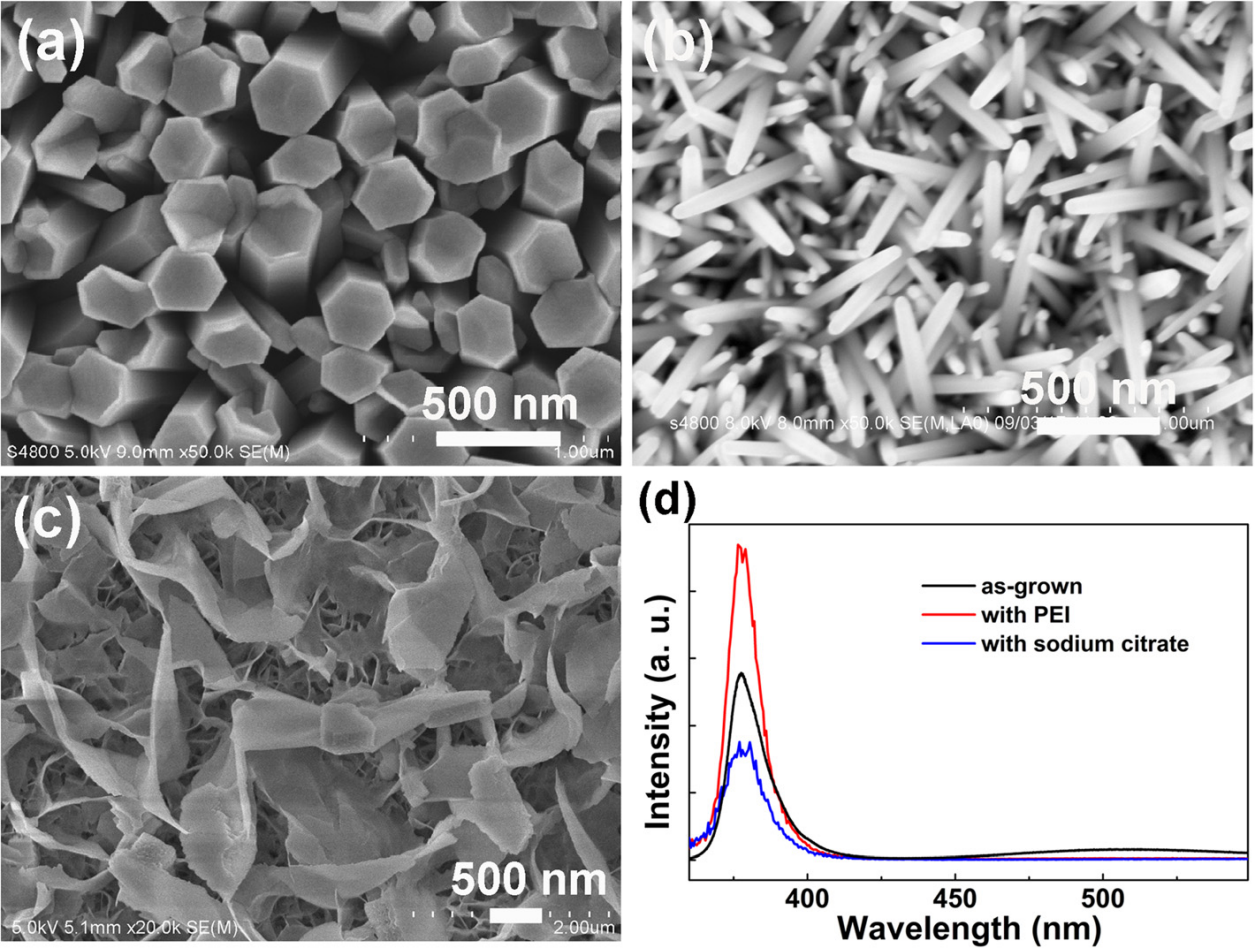
**Plan-view SEM images of ZnO nanostructures.** They are grown **(a)** without surfactants, **(b)** with 0.1 ml PEI, and **(c)** with 2.5 mg of sodium citrate (per 40 ml of reaction solution), at 0.05 M, 80°C for 5 h. **(d)** PL spectra of ZnO nanostructures in **(a)**, **(b)**, and **(c)**.

The crystal morphology can be tuned by introducing various surfactants, which could preferentially adsorb to different crystal faces, modifying the surface free energy and promoting (or suppressing) the growth along a certain direction [9,24]. High aspect ratio nanoneedles are possible to form by the introduction of an additive that suppresses radial growth but allows axial growth of the nanorods, such as polyethylenimine (PEI) and cetyltrimethylammonium bromide, while ZnO nanoplatelets are formed if a low concentration of sodium citrate is added into the reaction solution [24]. Figure 6a, b, c presents the plan-view SEM images of ZnO nanostructures grown without surfactants, with 0.1 ml PEI, and with 2.5 mg of sodium citrate (per 40 ml of reaction solution), respectively. As no surfactant is added, the average diameter of the ZnO nanorods is about 250 nm, which resulted from the rapid lateral growth at a high solution concentration. Introducing a proper amount of PEI into the reaction solution, the average diameter decreased sharply to about 60 nm; meanwhile, the as-grown ZnO nanorods turned into ZnO nanoneedles, as shown in Figure 6b. This should be contributed to the inhibited lateral growth by the adsorption of PEI on the lateral plane of the nanorods [1]. By adding a certain amount of sodium citrate, we can see from Figure 6c that ZnO nanoplatelets are obtained, which have potential application in sensors because of their large surface area.

It is well known that the optical properties of ZnO nanostructures are crucially dependent on their morphology. In addition, the optical properties of ZnO nanostructures would be improved due to surface passivation effects of polymer surfactants [27,28]. Thus, the PL measurements were performed to evaluate the optical quality of the obtained ZnO nanostructures, and the corresponding results were shown in Figure 6d. It can be seen that the PL spectrum of the ZnO nanorods grown with no surfactant exhibits a dominant UV emission at 377 nm, along with a weak visible emission around 520 nm. Generally, the UV emission is due to the near-band edge (NBE) emission of ZnO, and the visible emission can be attributed to intrinsic defects such as oxygen vacancies [29,30]. For the ZnO nanoneedles or platelets, grown with the addition of PEI or sodium citrate, the PL spectrum presents a unique UV emission (377 nm), but the defect-related visible emission is suppressed, which is attributed to the surface passivation effects of surfactants via the adsorption in different crystal faces and modification of the surface free energy. Furthermore, the intensity of NBE emission varies greatly with the morphology of ZnO nanostructures (nanorods, nanoneedles, or nanoplatelets), demonstrating that the photoluminescence property of ZnO nanostructures is adjusted by introducing different surfactants.

## Conclusions

In conclusion, the morphology evolution of the ZnO nanostructures was well monitored by tuning the hydrothermal growth parameters, such as seed layer, solution concentration, reaction temperature, and surfactant. It was found that both deposition methods and thickness of the seed layer could affect the orientation and morphology of the resulting ZnO nanorods; moreover, the length of ZnO nanorods depended mainly on the reaction temperature, while the diameter was closely related with the solution concentration. In addition, the morphology, as well as the optical properties, was tuned effectively by introducing various surfactants. The ease of synthesis, ability to control morphology, and optical properties make this approach promising in LEDs, sensors, and other applications.

## Abbreviations

AFM: Atomic force microscopy; NBE: Near-band edge; PEI: Polyethylenimine; PL: Photoluminescence; RF: Radio-frequency; rms: Root-mean-square; SEM: Scanning electron microscopy; XRD: X-ray diffraction.

## Competing interests

The authors declare that they have no competing interests.

## Authors’ contributions

J-JD designed the experiment, analyzed results, and participated in drafting the manuscript. C-YZ carried out the experiment, and X-WZ supervised the research and revised the manuscript. H-YH, JX, Z-LZ, and Z-YZ offered technical supports. All authors read and approved the final manuscript.
